# Species- and breed-associated heterogeneity in age-related increases in periodontal disease risk among dogs and cats based on Japanese insurance claim data

**DOI:** 10.3389/fvets.2026.1764413

**Published:** 2026-02-10

**Authors:** Noriyoshi Akiyama, Yuki Matsumoto, Ryo Horie

**Affiliations:** 1 Research and Developmental Division, Anicom Insurance, Inc., Yokohama, Kanagawa, Japan; 2 Research and Developmental Division, Anicom Specialty Medical Institute, Inc., Yokohama, Kanagawa, Japan; 3 Data Science Center, Azabu University, Sagamihara, Kanagawa, Japan

**Keywords:** age-related risks, body size, breed differences, breed groups, cats, dogs, insurance claim data, periodontal disease

## Abstract

**Introduction:**

Periodontal disease is one of the most common oral disorders in companion animals. However, quantitative evidence regarding whether the rate of age-related increases in risk differs among individual breeds, breed groups, or body size categories remains limited. This study aimed to characterize age-associated patterns and breed-group-level variations in periodontal disease among dogs and cats using nationwide insurance claims data from Japan.

**Methods:**

The analyses primarily used anonymized claims records from animals whose insurance policies began in 2023. After excluding breeds with fewer than 100 individuals, the dataset comprised 688,665 dogs representing 81 breeds and 185,782 cats representing 38 breeds. Generalized linear mixed models with a binomial distribution and logit links were applied.

**Results:**

For both species, the claims rate increased with age, and the rate of increase in cats was approximately 3.5% lower than that of dogs. In dogs, body size showed a strong association with the predicted probability of a periodontal disease claim at less than 1 year of age (baseline risk); however, the slopes of the age effect were similar across size categories. At the breed-group level, four epidemiological patterns were identified based on the combination of baseline risk and the magnitude of the age effect: (1) high baseline risk with a gentle slope, (2) low baseline risk with a steep slope, (3) moderately high baseline risk with a steep slope, and (4) both baseline risk and slope near the overall mean.

**Conclusion:**

No significant correlation was observed between baseline risk and age, suggesting that the initiation and progression of periodontal disease in dogs is governed by partially distinct determinants. In cats, the baseline risk showed minimal variation among breeds, whereas the effect of age differed. Brachycephalic breeds, including Exotic, Himalayan, and Persian cats, exhibit steep age-related increases in risk. These findings provide a comprehensive overview of age- and breed-related patterns of periodontal disease risk in dogs and cats using real-world data and highlight the importance of preventive dental care tailored to age and breed.

## 1 Introduction

Periodontal disease is a multifactorial inflammatory condition of the supporting structures of teeth, encompassing a continuum of pathological changes ranging from gingivitis to periodontitis (1–3). Although gingivitis can be reversed with appropriate treatment and oral hygiene, advanced periodontitis is irreversible and often leads to the destruction of the periodontal tissues (gingiva, alveolar bone, periodontal ligament and cementum), frequently resulting in tooth loss (4, 5).

Periodontal diseases are among the most common oral disorders (6–9). In both dogs and cats, periodontal disease affects quality of life and has been associated with systemic conditions such as cardiac, renal, and hepatic disorders, and even with increased mortality risk (9–14). Reported prevalence varies widely from approximately 9 to 80% in dogs (7, 14, 15) and 14 to 96% in cats (7, 16–18) mainly because of differences in diagnostic criteria and study populations. For example, in a prospective study with strict diagnostic criteria and a low clinical threshold, a prevalence of 56% was reported in a population of 1- to 6-years-old beagle dogs (19), whereas studies based on primary-care data reported prevalence estimates for mixed-breed dog populations of 12.5% in the United Kingdom (20) and 19.5% in the United States (6).

Several studies reported breed-specific differences in prevalence among dogs. West Highland White Terriers, Border Terriers, Toy Poodles, and Greyhounds are commonly affected, whereas German Shepherds and French Bulldogs show a relatively low prevalence (5, 7, 20, 21). Body weight (22) and cranial morphology (20) have also been implicated as contributing factors. In cats, some studies identified a higher prevalence in Persian, Maine Coon, and Siamese breeds (9, 18), whereas others found no significant breed effect (17, 23); thus, breed differences remain inconclusive.

Age-related increases in the occurrence and severity of periodontal disease have been repeatedly reported in both species. Harvey et al. (22) examined 1,350 North American dogs under anesthesia and found that approximately 15% of dogs over 10 years of age had furcation lesions in the maxillary fourth premolar teeth, with attachment loss and tooth loss increasing with age. Similarly, analyses of primary-care datasets in the United States (6) and the United Kingdom (20) showed that prevalence rose with age, with dogs aged ≥8 years having about three times the odds of periodontal disease compared with those aged 2–4 years. A longitudinal study on Miniature Schnauzers also confirmed the progressive worsening of periodontal status with increasing age, identifying aging as a major driver of disease progression (21). A standard-colony study of 109 cats demonstrated that older mixed-breed cats exhibited significantly higher periodontal inflammation scores compared to younger cats (18), and a large-scale analysis of the VetCompass dataset found that cats aged 9–12 years had approximately six to seven times the odds of disease compared with those aged <3 years (9). Furthermore, Niemiec (24) estimated that approximately 80% of adult dogs and 70% of adult cats over 2 years of age are affected, supporting the notion that periodontal disease is highly prevalent among mature companion animals. However, the extent to which the rate of age-related increases in prevalent risk varies among body sizes, breed groups, and individual breeds is rarely investigated and remains largely unknown. Identifying breeds or groups that show a steep increase in prevalent risk with aging despite low baseline prevalence could provide valuable insight for more individualized preventive and clinical strategies.

This study was designed to investigate age-related patterns in periodontal disease morbidity among dogs and cats using net insurance claims data. Insurance data provide diagnostic information from many veterinary clinics, reflecting real-world clinical practice. This study analyzed data from Anicom Insurance Inc. (Tokyo, Japan), Japan’s largest pet insurance company. Although the overall pet insurance enrollment rate in Japan is approximately 15%, which is lower than that in Sweden (around 80%) according to 2023–2024 international statistics, the Anicom database covers a broad and representative population across Japan with minimal geographic and clinic-level bias because participating veterinary clinics are distributed nationwide. Small-breed dogs are particularly common in Japan (25). Therefore, this dataset is well-suited for examining the effects of aging, body size, and breed on the prevalence of periodontal disease.

## 2 Materials and methods

### 2.1 Ethical considerations

All data analyses were conducted under Anicom Insurance, Inc.’s data use policy and in accordance with the relevant privacy protection regulations. The use of the data was covered by the policyholders’ consent obtained at the time of insurance enrollment, which allowed the secondary use of anonymized claim information for research and statistical purposes. Individual policy numbers were used only to identify eligible contracts during data extraction and were removed before analysis; thus, no personal identifiable information was included in the research dataset. Ethical review by an institutional committee was not required for the secondary analysis of anonymized data.

### 2.2 Data source and study population

The data used in this study were obtained from Anicom Insurance Inc.’s pet insurance claims database. This database contains information on insured dogs and cats, including species, breed, sex, age, policy inception date, and diagnostic terms for clinical conditions, at two hierarchical levels: major and minor. The major diagnostic category represents broad disease groups (e.g., diseases of the teeth and oral cavity, ocular diseases, and cardiac diseases), whereas the minor diagnostic term indicates specific conditions (e.g., periodontal disease, gingivitis, cataract, and hypertrophic cardiomyopathy). These diagnostic terms were entered by veterinarians based on clinical records, either by selection from a standardized list or by entering free-text descriptions. Because diagnoses were made in routine clinical practice across multiple veterinary hospitals, the specific diagnostic criteria for each condition may have varied among veterinarians and institutions. Claims related to preexisting conditions diagnosed before policy inception were excluded because such conditions were not covered under the insurance policy. The following policies were also excluded: (1) those without outpatient coverage, (2) those with a policy term other than 1 year, and (3) those who canceled immediately after enrollment due to a cooling-off request or similar reasons. Each year was treated as a single observation per animal. Analyses were conducted using claims data from policies commenced in 2013, 2018, and 2023. Animals with missing information regarding sex, age, or breed were excluded from the analysis. Sex was recorded as male or female only, and information on neuter status was not available. Crossbred animals, including crosses between different pure breeds as well as Japanese mixed-breed dogs and cats, were registered under the unified categories “mixed-breed dog” or “mixed-breed cat” and were excluded from our analyses. The numbers of breeds and animals included in the initial datasets were as follows: for dogs, 183 breeds (*n* = 410,506) in 2013, 178 (*n* = 551,871) in 2018, and 183 (*n* = 690,687) in 2023 (Supplementary Table S1); for cats, 51 breeds (*n* = 39,432) in 2013, 62 (*n* = 90,434) in 2018, and 68 (*n* = 186,605) in 2023 (Supplementary Table S2). Data from 2013 and 2018 were used to examine temporal trends. Detailed analyses were performed using the 2023 dataset, which contained the largest number of records.

### 2.3 Definition of variables and grouping

For each animal, a binary indicator of periodontal disease-related morbidity was assigned based on the presence or absence of insurance claims related to periodontal conditions. Animals were classified as positive for periodontal disease if the minor diagnostic term in the claims record contained any of the following terms: “periodontal disease,” “gingivitis,” “alveolar pyorrhea,” “dental root abscess,” or “periapical abscess.” The search was conducted by partial text matching of these Japanese terms, encompassing both standardized diagnostic entries and free-text descriptions. Animals without any of these terms were considered negative. The number of animals identified for each diagnostic term is summarized in Supplementary Table S3.

For dogs, two breed-level categorical variables were included: body size and breed. Body size groups were classified into five categories based primarily on reference adult body weight for each breed: Toy (<4 kg), Small (4–<10 kg), Medium (10–<30 kg), Large (30–<45 kg), and Giant (≥45 kg). The reference body weight for each breed provided by the Japan Kennel Club (JKC). The breed groups were defined according to the 10-group classification of the Fédération Cynologique Internationale (FCI) as adopted by the JKC: G1, Sheepdogs and Cattledogs; G2, Pinscher and Schnauzer – Molossoid and Swiss Mountain and Cattledogs; G3, Terriers; G4, Dachshunds; G5, Spitz and primitive types; G6, Scent hounds and related breeds; G7, Pointing dogs; G8, Retrievers – Flushing dogs – Water Dogs; G9, Companion and Toy dogs; and G10, Sighthounds. Breeds for which group information could not be assigned were excluded from the group-based analyses. For cats, neither body size nor breed classification was applied, and the analyses were conducted only at the breed level.

### 2.4 Analytical overview

The analyses were performed in four sequential phases based on generalized linear mixed models (GLMMs) with a binomial error distribution and a logit link. First, using policy records initiated in 2013, 2018, and 2023, temporal changes in age-specific periodontal disease claim rates were evaluated separately for dogs and cats. Second, data from 2023 were used to compare the overall morbidity risk and the magnitude of age-related effects (per-year change in the odds of a periodontal disease claim) between the two species. Third, within the 2023 dog dataset, baseline risk (probability of a periodontal disease claim at age zero) and age-related effects were compared among body size categories and breed groups (JKC/FCI classification) to examine whether body size or phylogenetic grouping contributed to the observed variation. Finally, breed-level models were fitted using the data from 2023 to quantify the baseline risk and age-related increase for each breed, evaluate the correlation between these two parameters, and identify breeds with distinctive characteristics in age-related periodontal disease risk.

### 2.5 Statistical analysis

The analyses were conducted in R (v4.3.3; R core Team, 2024) using GLMMs with a binomial error distribution and a logit link [lme4 (26)::glmer, Laplace approximation; optimizer = “bobyqa”]. To ensure stable estimates, breeds with fewer than 100 individuals were excluded from the analyses using the 2023 dataset (dogs: 81 breeds, *n* = 688,665; cats: 38 breeds, *n* = 185,782). Unless otherwise noted, Wald-type confidence intervals and tests were used for fixed effects, and results are presented as odds ratios (ORs) with 95% confidence intervals (CIs). Sex was coded as a binary factor (male, reference). Statistical significance was defined as *p* < 0.05 (two-sided) unless otherwise stated. Plots were generated using R packages ggplot2 (27), gridExtra (28), cowplot (29), and scales (30).

#### 2.5.1 Annual comparisons (dogs and cats)

To assess temporal differences in morbidity (policy start years: 2013, 2018, and 2023), we fitted species-specific GLMMs, including age at policy inception (continuous), sex, and start year (categorical) as fixed effects, with a random intercept for breed. Breed was modeled as a random effect to appropriately account for systematic differences in baseline risk among breeds, which can arise from genetic background, craniofacial and dental morphology, or breed-related lifestyle factors. Given the large number of breeds included in the dataset and the substantial imbalance in sample size across breeds, treating the breed as a random effect also enabled partial pooling, preventing unstable parameter estimates that would occur if each breed was modeled as a fixed effect. This approach allowed us to absorb breed-level heterogeneity while obtaining more robust estimates of the main effects of age, sex, and year. The model formula was:


$$\begin{array}{l} \text{logit}\{\mathrm{Pr}(\text{periodontal claim})\}=\beta_{0}+\beta_{1}(\mathrm{age})+\beta_{2}(\mathrm{sex})\\ +\beta_{3}(\text{start year})+b_{0,\text{breed}} \end{array}$$

The choice between the GLMM and a corresponding generalized linear model (GLM) without random effects was guided by Akaike’s Information Criterion (AIC). Given the boundary nature of random-effect variances, we did not rely on naïve likelihood ratio tests for random-effect presence. When needed, fixed-effect contrasts were evaluated using Wald tests.

#### 2.5.2 Dog–cat comparison in 2023

Using 2023 data, we compared the species by fitting a GLMM with an age-by-species interaction (plus sex) and a random breed intercept:


$$\begin{array}{l} \text{logit}\{\mathrm{Pr}\}=\beta_{0}+\beta_{1}(\mathrm{age})+\beta_{2}(\text{species})\\ +\beta_{3}(\mathrm{sex})+\beta_{4}(\mathrm{age}\times \text{species})+b_{0,\text{breed}} \end{array}$$

The interaction term tested whether the annual OR differed between dogs and cats.

#### 2.5.3 Dog size and breed groups by 2023

Within the 2023 dog dataset, we examined the heterogeneity across body size categories and breed groups to determine whether body size or phylogenetic grouping contributed to the observed variation in periodontal disease risk. Dogs lacking information on either body size category or breed group were excluded. However, all the dogs were removed during the filtering step, excluding breeds with fewer than 100 individuals. Therefore, no additional exclusions were made during this stage. For each classification (body size and breed group), we fitted GLMMs, including an interaction between age and size category (or breed group), sex as a covariate, and a random intercept for breed to account for breed-level heterogeneity:


$$\begin{array}{l} \text{logit}\{\mathrm{Pr}\}=\beta_{0}+\beta_{1}(\mathrm{age})+\beta_{2}(\text{category})\\ +\beta_{3}(\mathrm{sex})+\beta_{4}(\mathrm{age}\times \text{category})+b_{0,\text{breed}} \end{array}$$

The primary hypothesis in each model was the equality of age slopes across categories or groups. Estimated baseline morbidity (predicted probability at age 0) and per-year ORs (age effects) with Wald 95% CIs were derived from the fixed effects and conditional variances of the random effects. Pairwise comparisons of the per-year ORs across categories or groups were conducted with Tukey’s adjustment for multiplicity using the emmeans package (31).

#### 2.5.4 Breed-level models (2023)

To quantify the breed-specific baseline risk and age-related increases, we fitted GLMMs with random intercepts and random slopes for age by breed.


$$\begin{array}{l} \text{logit}(\mathrm{Pr})=\beta_{0}+\beta_{1}(\mathrm{age})+\beta_{2}(\mathrm{sex})\\ +b_{0},\text{breed}+b_{1},\text{breed}(\mathrm{age}) \end{array}$$

For dogs, this allowed the estimation of (i) breed-specific baseline log-odds at age 0 and (ii) breed-specific age slopes, corresponding baseline morbidity (predicted probability at age 0), and per-year ORs (age effect). Wald 95% CIs were derived from the fixed effects and conditional variances of the random effects. For cats, the variance components indicated negligible between-breed variance in baseline risk. Breed-level baseline estimates were, therefore, treated as homogeneous, whereas random slopes for age were retained to summarize the variability in age-related increases.

## 3 Results

### 3.1 Year-wise comparisons (dogs and cats)

**Figure 1:**
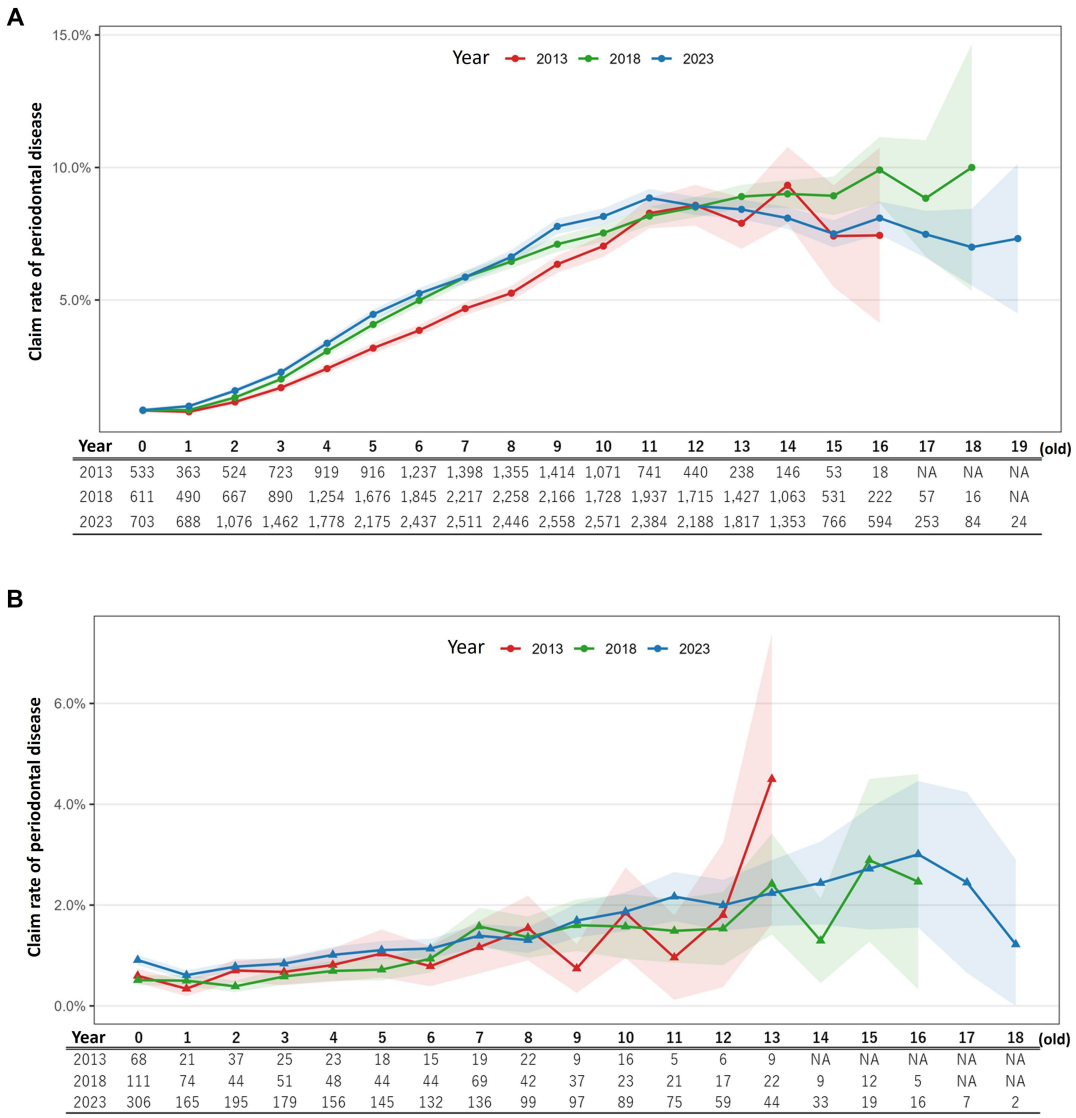
Age-specific periodontal disease claim rates by policy year. The denominator includes all insured animals, including those without submission of claims (i.e., animals that did not visit a veterinary clinic during the policy year). Solid lines represent age-specific variations in periodontal disease claim rates, with each color indicating a different policy year. Shaded areas in corresponding lighter colors represent 95% confidence intervals. The table below the plot shows the number of animals with periodontal-disease-related claims. Age categories with fewer than 100 claims events are omitted because the 95% confidence intervals became excessively wide. **(A)** Dogs. **(B)** Cats.

Using insurance policy data from 2013, 2018, and 2023, temporal changes in the occurrence of periodontal disease claims were evaluated separately for dogs and cats. The overall claim rates related to periodontal disease were as follows: dogs—2013: 2.95% (12,093/410,506), 2018: 4.13% (22,774/551,871), and 2023: 4.33% (29,884/690,687); cats—2013: 0.76% (300/39,432), 2018: 0.75% (677/90,434), and 2023: 1.05% (1,956/186,605). When limited to individuals that had at least one insurance claim (i.e., animals that received veterinary care during the policy year), the corresponding rates were: dogs—2013: 4.62% (12,093/261,644), 2018: 6.40% (22,774/355,771), and 2023: 6.55% (29,884/456,464); cats—2013: 1.59% (300/18,865), 2018: 1.66% (677/40,782), and 2023: 2.28% (1,956/85,795). Model comparisons indicated that GLMMs with a random intercept for breed provided a better fit than did GLMs without random effects in both species (dogs: AIC 505,620 vs. 517,048; cats: AIC 32,719 vs. 32,767). Therefore, subsequent analyses were based on the GLMMs to account for breed-level heterogeneity. In dogs, the fixed effects (age at policy inception, sex, and policy start year) were statistically significant (all *p* < 0.001). The estimated ORs with 95% CI were as follows: age per year, 1.156 (1.153–1.158); female vs. male, 1.083 (1.066–1.100); and policy year 2018 and 2023 vs. 2013, both approximately 1.13 (2018: 1.129, 2023: 1.131). Age-specific claim rates and the number of claim events at each age are illustrated in Figure 1A (dogs) and Figure 1B (cats). These results indicated that the risk of periodontal disease claims in dogs increased with age, was slightly higher in females, and has risen modestly in recent years compared to 2013. In cats, age and sex were also significant predictors (*p* < 0.001), whereas policy-year effects were significant only for 2023. The OR for age per year was 1.105 (1.095–1.115), and females had a lower risk (OR 0.852, 0.791–0.917). The 2018 policy year showed no significant difference compared with 2013, whereas the 2023 cohort exhibited a 1.238-fold higher risk (1.094–1.402). Thus, as in dogs, the risk of periodontal disease claims in cats increases with age and appears to be higher in the most recent period, although the effect of sex is in the opposite direction. The variance of the random intercept for breed was 0.49 [standard deviation (SD) = 0.70] in dogs and 0.05 (SD = 0.22) in cats, indicating that breed-level heterogeneity was more pronounced in dogs.

### 3.2 Dog–cat comparison (2023 data)

**Figure 2:**
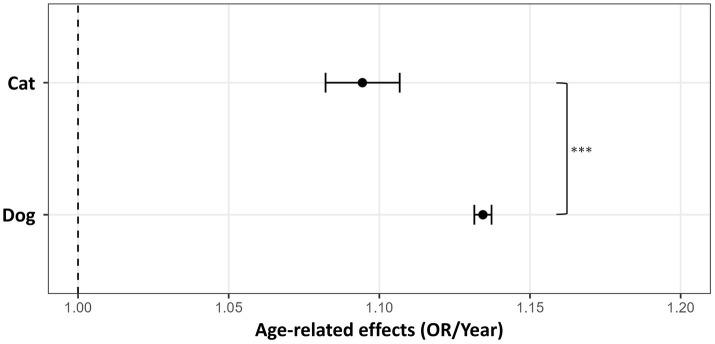
Age-related increase in periodontal disease risk in dogs and cats. Data from 2023 cohort were used for comparison. The dotted line at 1.00 represents no age-related change. Error bars indicate 95% confidence intervals. *** denotes *p* < 0.001.

Using insurance policy data from 2023, we compared the risks of periodontal disease-related claims and age-related changes between dogs and cats. A GLMM was applied with age, species, and sex as fixed effects and breed as a random effect. All fixed effects were statistically significant (*p* < 0.001). In dogs, the age coefficient was 0.126 (OR = 1.134, 95% CI: 1.132–1.137), indicating that the risk of periodontal disease–related claims increased by approximately 13.4% per year of age (Figure 2). For cats, the age-by-species interaction term (age × cat) had a coefficient of −0.036, corresponding to an OR of 0.965 (95% CI: 0.954–0.976). This result indicated that the age-related increase in risk was approximately 3.5% per year smaller in cats than in dogs, and the difference in age effects between species was statistically significant (*p* < 0.001). The age effect for cats, calculated as the sum of the coefficients for age and the interaction term (0.126 + −0.036 = 0.090), corresponded to an OR of 1.094 (95% CI: 1.082–1.100) (Figure 2). Thus, although the risk of periodontal disease-related claims increased with age in both species, the rate of increase was more gradual in cats. Additionally, at baseline (age 0), cats had a significantly lower risk (OR = 0.538, 95% CI: 0.428–0.675). These results suggest that, in addition to the effect of age, cats have a lower initial risk of periodontal disease than do dogs.

### 3.3 Analyses by category in dogs

#### 3.3.1 Analysis by body size

**Figure 3:**
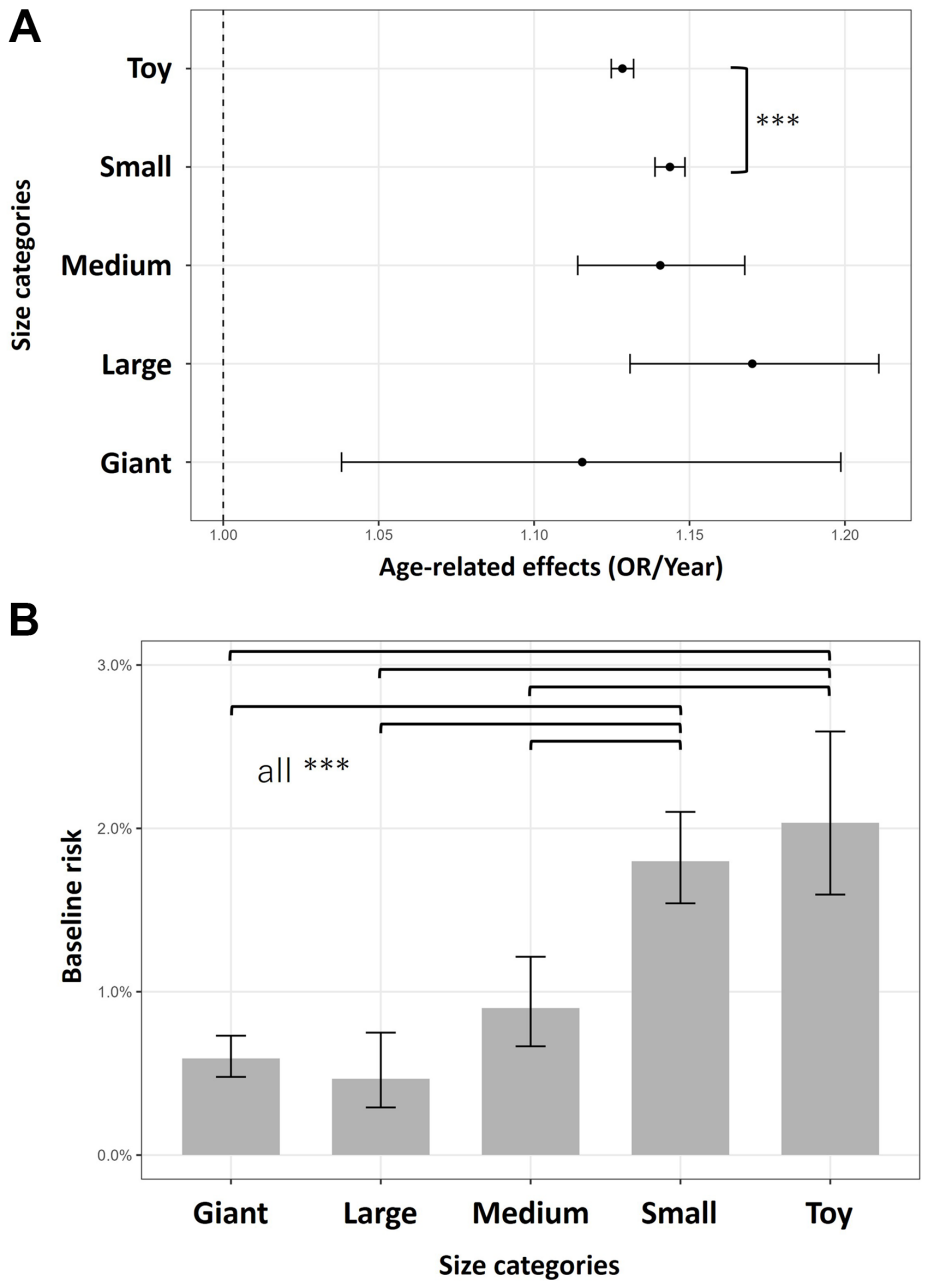
Periodontal disease risk across dog body-size categories. Error bars indicate 95% confidence intervals. Tukey-adjusted *p*-values were used for multiple comparisons, and *** denotes *p* < 0.001. **(A)** Differences in age-related effects across size categories. The dotted line at 1.00 represents no age-related change. **(B)** Differences in baseline risk differences across size categories. Significant differences were observed in all pairwise comparisons between Small/Toy and Giant/Large/Medium breeds.

Based on the 2023 dataset, the risk of periodontal disease-related claims and age-related effects of periodontal disease claims were compared among the body size categories (Giant, Large, Medium, Small, and Toy). The number of dogs in each category was as follows: Giant, 3,025; Large, 11,607; Medium, 24,095; Small, 253,849; and Toy, 396,089, totaling 689,000 dogs (81 breeds). A GLMM including age, body size, the interaction of age × body size, and sex as fixed effects, and breed as a random intercept was applied. All the fixed effects (joint test: *p* < 0.001) and the interaction of age × body size (chi-sq = 28.640, *p* < 0.001) were significant, indicating that the rate of age-related increases in claim risk differed slightly among body size categories. The annual ORs (OR per year, 95% CI) of periodontal disease claims were as follows: Giant, 1.115 (1.038–1.199); Large, 1.170 (1.131–1.211); Medium, 1.141 (1.114–1.168); Small, 1.144 (1.139–1.149); and Toy, 1.128 (1.125–1.132) (Figure 3A). All the categories showed an age-related increase in risk ranging approximately from +11% to +17% per year. Post-hoc comparisons (Tukey-adjusted) revealed a significant difference only between the Small and Toy categories (*p* = 5.8 × 10^−6^, ratio of OR/year = 1.014). The difference in the slope between the two groups was 1.4% per year, and no other pairwise differences were significant. Thus, even substantial contrasts in body size (e.g., toy vs. giant breeds) were not associated with detectable differences in age-related effects.

The baseline risk (corresponding to the model intercept) also differed significantly among the body size groups (joint test: chi-square = 75.128, *p* < 0.001). Pairwise Tukey-adjusted comparisons showed that the Giant, Large and Medium groups had significantly lower baseline risks than did the Small and Toy groups (all p < 0.001). No significant differences were observed between the Small and Toy groups (Figure 3B). These results indicated that smaller breeds, particularly Small and Toy dogs, had much higher baseline risks (over three times greater than that of giant breeds), whereas the rate of age-related increase in risk was similar across all the body size groups.

#### 3.3.2 Analysis by breed group

**Figure 4:**
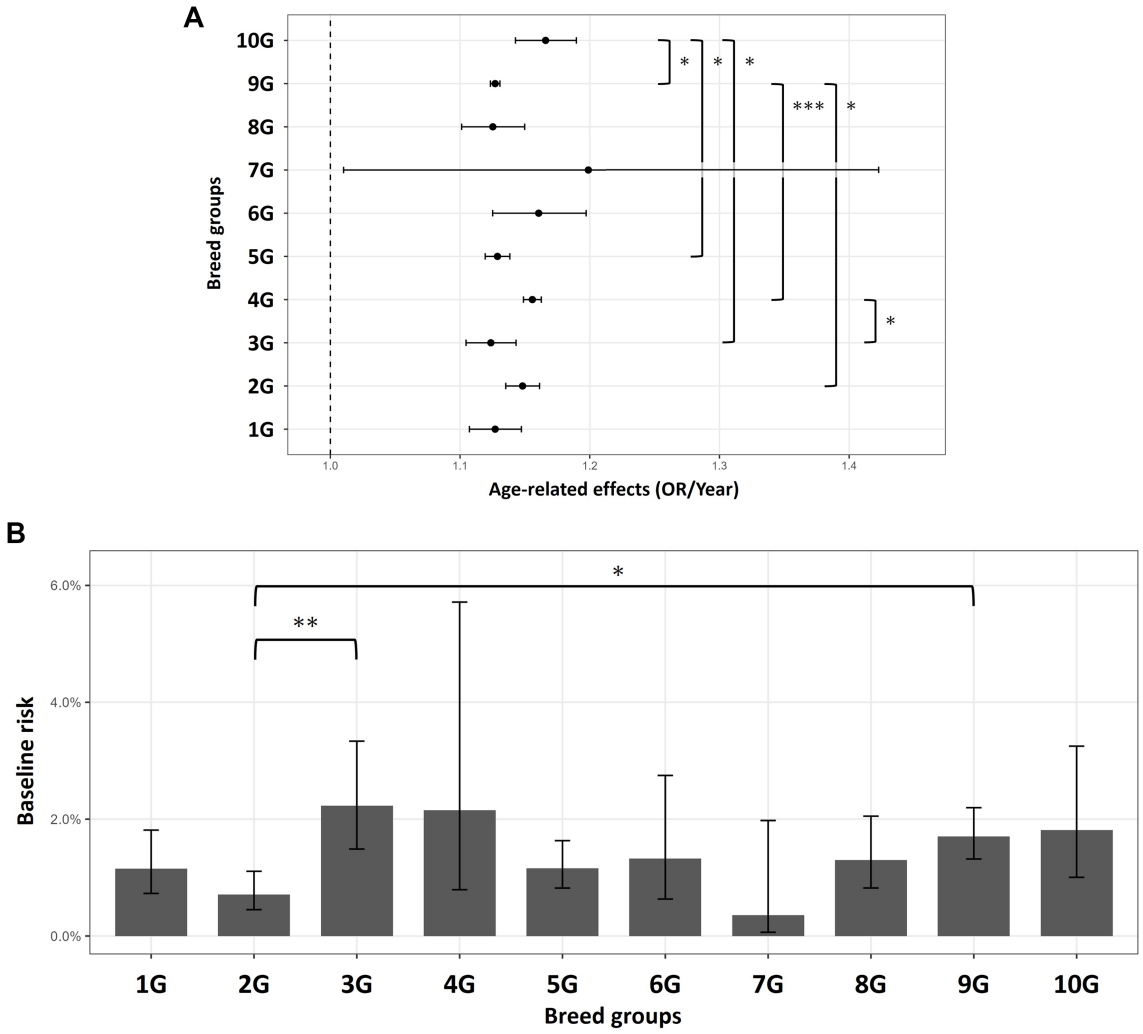
Periodontal disease risk across dog breed groups. Error bars indicate 95% confidence intervals. Multiple comparisons were performed using false discovery rate. * denotes *p* < 0.05, ** denotes *p* < 0.01, and *** denotes *p* < 0.001. **(A)** Differences in age-related effects among the breed groups. The dotted line at 1.00 represents no age-related change. **(B)** Differences in baseline risk among the breed groups.

Next, the differences in the risk and age-related effects of periodontal disease claims were analyzed among the 10 breed groups classified by the JKC. The number of dogs in each group was 1G (sheep and cattle): 20,010, 2G (Working Dogs): 36,073, 3G (Terriers): 11,203, 4G (Dachshunds): 71,778, 5G (Spitz and Primitive Types): 105,056, 6G (Scent Hounds): 5,670, 7G (Pointers and Setters): 556, 8G (Retrievers, Flushing, and Water Dogs): 22,479, 9G (Companion): 409,221, and 10G (Sighthounds): 6,619. The joint test revealed a significant interaction between age and breed (chi-square = 70.173, *p* < 0.001). All the groups exhibited an age-related increase in the risk of periodontal disease claims; however, the magnitude of this effect varied slightly among the groups. The estimated age-related ORs (OR per year, 95% CI) were 1G: 1.127 (1.107–1.147), 2G: 1.148 (1.135–1.161), 3G: 1.124 (1.105–1.143), 4G: 1.156 (1.149–1.163), 5G: 1.129 (1.119–1.138), 6G: 1.161 (1.125–1.197), 7G: 1.199 (1.010–1.420), 8G: 1.125 (1.101–1.150), 9G: 1.127 (1.123–1.131), and 10G: 1.166 (1.143–1.190). Groups with relatively high age effects included 4G, 6G, 7G, and 10G, whereas those with milder slopes included 1G, 3G, 5G, 8G, and 9G (Figure 4A). As multiple pairwise comparisons were conducted, *p*-values were adjusted using the false discovery rate (FDR) method. After FDR adjustment, significant differences were found between the 9G and 10G (*p* = 0.017), 2G (*p* = 0.022), and 4G (*p* < 0.001) groups. Additionally, 10G showed a significantly stronger age effect than did 3G (*p* = 0.040) and 5G (*p* = 0.028), whereas 4G showed a stronger effect than did 3G (*p* = 0.022). These differences were at most approximately 3–4% per year.

The baseline risk also differed among the groups (joint test: chi-square = 24.093, *p* = 0.0042). According to the FDR-adjusted pairwise comparisons, 2G (Working Dogs) had a significantly lower baseline risk than did 3G (*p* = 0.008) and 9G (*p* = 0.023). This finding indicated that Working Dogs had the lowest baseline risk of periodontal disease claims, whereas Companion Dogs and Terriers had relatively higher baseline risks (Figure 4B). Although not significantly different from other groups, the baseline risk of 4G (2.15%, 95%CI: 0.79–5.71) and 10G (1.81%, 95%CI: 1.00–3.25) was as high as 3G (2.23%, 95%CI: 1.49–3.33) and 9G (1.70%, 95%CI: 1.32–2.20). Overall, the results show that the risk of periodontal disease claims increased with age across all the groups, with some groups exhibiting higher baseline risks and steeper age-related effects.

### 3.4 Breed-level analysis

Using the 2023 dataset, the breed-level risks and age-related effects of periodontal disease claims were evaluated. For both dogs and cats, a GLMM with a logistic link was applied, including age and sex as fixed effects, and breed as a random effect. Random intercepts and random slopes for age were specified to simultaneously estimate the breed-specific baseline risk and age-related effects.

**Figure 5:**
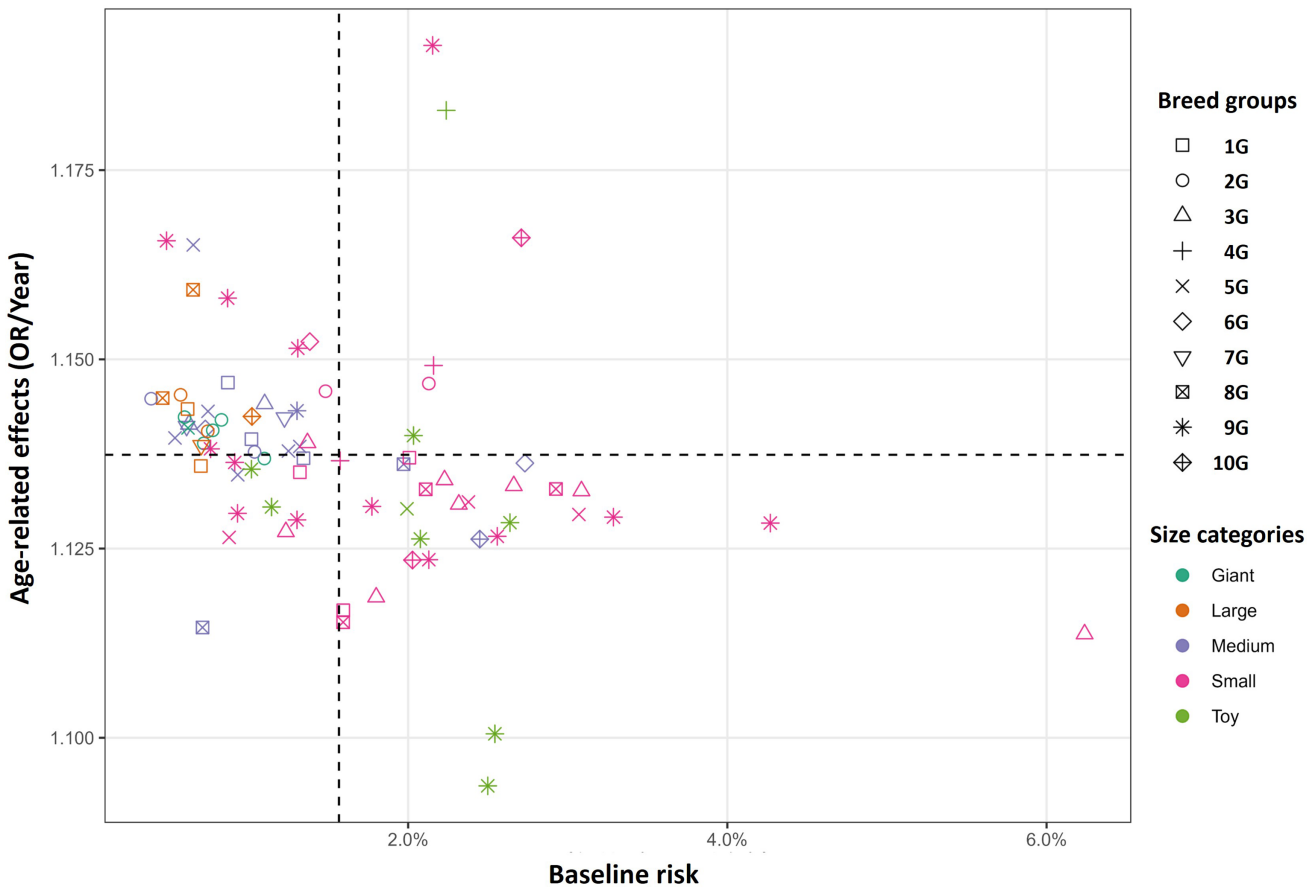
Breed-specific patterns of two periodontal disease risk indices in dogs. Dotted lines represent mean values for each axis. Marker shapes differed by breed group, and colors differed by body size category. Confidence intervals are omitted for clarity but are provided in Supplementary Table S4. The correlation coefficient between baseline risk and age-related effects was −0.34; however, a likelihood ratio test comparing the original model with a model assuming zero correlation yielded *p* = 0.17, indicating that the correlation was not statistically significant.

In dogs, the variance of the random effects was 0.457 (SD = 0.676) for the intercept (baseline risk) and 0.00051 (SD = 0.0225) for the age slope, with a correlation coefficient of −0.34 between them. Thus, the baseline risk varied considerably among breeds, whereas the variation in the age-related effect was small, and the correlation between the two components was not statistically significant. A likelihood ratio test comparing the full model with one model, assuming zero correlation, showed no significant differences (*p* = 0.17). A two-dimensional plot of breed-specific baseline risk versus age-related effects is shown in Figure 5. Small-sized breeds generally exhibit higher baseline risks and lower age-related effects, although some breeds exhibit uniquely strong age-related effects or markedly lower baseline risks. Overall, the breeds could be broadly divided into those with a high baseline risk from an early age (e.g., companion and terrier-type breeds) and those showing a marked increase in risk with aging (e.g., working and hound-type breeds). Estimated breed-specific baseline risks and age-related effects, with 95% CIs for all the dog breeds, are shown in Supplementary Table S4.

In cats, the variance in the random effects was 0 (SD = 0.000) for the intercept and 0.00063 (SD = 0.0250) for the age slope, indicating minimal variation in the baseline risk among breeds and only a slight variation in age-related effects. Accordingly, a correlation between baseline risk and age-related effects could not be estimated. Based on the magnitude of age-related effects, five breeds with the highest and lowest age-related effects were identified (Supplementary Figure S1). The breed with the strongest age-related effect was the Siamese (OR per year = 1.144), whereas the breed with the weakest effect was the Russian Blue (OR per year = 1.056). The estimated age-related effects (OR per year) with 95% CIs for all the cat breeds are summarized in Table S5. Because all cat breeds share the same baseline risk, Table S5 does not include the baseline risk estimates.

## 4 Discussion

### 4.1 Summary of main findings

This nationwide large-scale analysis based on Japanese insurance claims data identified a clear age-related increase in the risk of periodontal disease in both dogs and cats, capturing consistent age-related patterns across species and analytical levels.

In dogs, both the magnitudes of the age effect and baseline risk varied substantially among body size categories, breed groups, and individual breeds, whereas in cats, the variation in baseline risk among breeds was minimal.

### 4.2 Temporal trends in insurance claims

Among insured animals that received veterinary care, the claim rates related to periodontal disease (dogs: 4.62–6.55%; cats: 1.59–2.28%) were lower than the prevalence estimates reported in primary-care studies [dogs—United States: 19.5% (6), United Kingdom: 12.5% (20); cats—United Kingdom: 15.2% (9)]. Although a lower prevalence of periodontal disease in the Japanese pet population cannot be completely excluded, the relatively low claim rates observed here are more likely to be attributable to the structural characteristics of the insurance data. Insurance claim records are based on veterinary clinical diagnoses but are generated only when the diagnosis or treatment is submitted for reimbursement. Consequently, cases in which only uncovered procedures (e.g., prophylactic scaling) were performed or mild gingivitis without treatment may not have been recorded. In a population of Japanese dogs, even among elderly individuals, gingivitis accounted for 66.7% of cases, whereas periodontitis accounted for only 24.0% (32). The fact that most animals diagnosed with periodontal disease present with gingivitis further supports the interpretation that insurance-based data likely underestimate the true population prevalence of periodontal disease. The veterinarians’ diagnostic awareness and sensitivity to periodontal disease may have also influenced these results. In both dogs and cats, the claims rate for periodontal disease in 2023 was significantly higher than that in 2013. As no major changes occurred in the insurance system during this period, this increase is unlikely to reflect a true nationwide surge in disease occurrence over only a few years but rather a heightened recognition and diagnostic sensitivity among veterinarians following increased scientific attention to periodontal disease. Furthermore, the higher prevalence of periodontal disease often reported in prospective clinical studies than in primary-care datasets is thought to result from lower diagnostic thresholds and the use of more rigorous oral examinations (20). As awareness of the importance of dental care continues to increase among veterinarians, insurance claim rates for periodontal diseases may increase in the future.

### 4.3 Species differences in age-related risk

In the 2023 comparison between dogs and cats, both species showed age-related increases in risk; however, the baseline risk and magnitude of the effect of age were higher in dogs. The baseline risk in cats was approximately half that in dogs (0.54-fold lower), and the slope of the age effect differed by 3.5% (dogs: OR/year = 1.134; cats: OR/year = 1.094), indicating that the disparity in periodontal disease claims rates between the two species widened with age. In contrast, primary-care studies in the United Kingdom reported an annual prevalence of 12.5% in dogs and 15.2% in cats, suggesting a relatively higher prevalence in cats (9, 20). This discrepancy may reflect the lower veterinary visitation rates among cat owners in Japan. According to a 2024 national survey, the number of cats owned (approximately 9.16 million) will exceed that of dogs (approximately 6.80 million) (33). However, in the present dataset, the number of insured cats was less than one-third of insured dogs, indirectly suggesting a lower rate of insurance enrollment, implying that healthcare utilization is lower in cats than in dogs. Furthermore, cats often exhibit behavioral tendencies to conceal signs of illness or pain, which may reduce the likelihood of visiting veterinary clinics. These social and behavioral factors may have contributed to the underestimation of the baseline risk in cats. However, these factors alone do not sufficiently explain the milder effect of age on periodontal disease in cats than in dogs. Biological and dental structural differences are likely to play a major role. Compared with dogs, cats possess shorter shear-type dentition and chew for a shorter duration, resulting in less crowding and fewer occlusal fractures. Periodontal disease arises primarily from the accumulation of dental plaque (4, 24), and in humans, alterations in oral pH have been associated with disease progression (34, 35). Cats have adapted to eating small meals frequently throughout the day, reflecting their natural hunting and feeding behavior (36). Although direct evidence is lacking for cats, these feeding patterns may influence the dynamics of oral pH fluctuations, thereby affecting plaque accumulation. These biological characteristics may partially account for the milder effects of age observed in cats.

### 4.4 Breed- and size-specific determinants of periodontal disease and their practical implications

For dogs, detailed analyses were conducted across three hierarchical levels: body size, breed group, and individual breed. At the body size level, smaller breeds showed distinctly higher baseline risks than did larger breeds, whereas the slope of the age-related increase did not differ substantially among size categories. This finding agrees with previous reports that identified small breeds as particularly prone to periodontal disease (20, 22) and suggests that although small dogs are predisposed to developing the disease at younger ages, their subsequent rate of progression with aging is comparable to that of larger dogs. This predisposition in small and toy breeds may be explained by anatomical characteristics, including a reduced jaw size relative to tooth number, which results in increased dental crowding and greater plaque retention in the oral cavity. At the breed-group level, both the baseline risk and age-related effects differed significantly, revealing distinct patterns among the groups. These results indicate that body size mainly influences baseline risk, whereas breed group affects both baseline risk and the magnitude of age-related increase. At the individual breed level, a weak negative correlation (r = −0.34) was observed between baseline risk and age-related effects, although this was not statistically significant, suggesting that the two components may vary independently. In other words, breeds that are at a high risk of developing periodontal disease early in life do not necessarily exhibit faster progression with aging. This tendency could partly reflect clinical and epidemiological factors such as earlier intervention in high-risk breeds or survival bias among severely affected individuals. Overall, these findings suggest that the initiation and progression of periodontal disease in dogs is governed by distinct determinants. Anatomical factors, such as body size and oral conformation, may strongly affect baseline risk, whereas breed-group characteristics, immune function, and lifestyle factors may contribute to age-related increases. Accordingly, effective periodontal disease management in dogs should consider not only age and body size but also the specific breed-group characteristics and inherent predispositions of each breed. Moreover, the risk patterns identified in this study may have practical relevance depending on the level of awareness among pet owners regarding the importance of dental and oral diseases. For example, in breed groups with high baseline risk, emphasizing the importance of early intervention from a young age may be beneficial, whereas in groups with strong age-related effects, communicating the need for continuous lifelong dental care is likely to be important. Such tailored communication based on body size and breed-group-specific risk profiles may help insurers and veterinary clinics promote beneficial behavioral changes among owners and contribute to the improved prevention and management of periodontal disease.

### 4.5 Breed-group patterns and breed-specific deviations

The analysis of breed groups revealed four distinct epidemiological patterns in the age-related trajectory of periodontal disease risk based on the combination of baseline risk and magnitude of the age effect: (1) High baseline risk with mild-to-moderate age-related increase [companion dogs (9G) and terriers (3G)]; (2) Low-to-moderate baseline risk with relatively steep age-related increase [working dogs (2G), scenthounds (6G), and pointers and setters (7G)]; (3) Moderately high baseline risk with steep age-related increase [dachshunds (4G) and sighthounds (10G)]; and (4) Both baseline risk and age-related increase near the overall mean [herding and cattledogs (1G), spitz-type breeds (5G), and retriever-type breeds (8G)]. These patterns suggest that the onset and progression of periodontal disease are influenced by distinct combinations of morphological, behavioral, and genetic factors. For instance, companion and terrier groups, which mainly consist of small breeds, may have early onset periodontal disease due to dental crowding and smaller oral cavity volume but show relatively moderate progression with age. In contrast, large-breed groups, such as working dogs and hounds, may have a low risk at young ages but experience a steeper increase later in life, possibly owing to stronger occlusal forces, tooth wear, and other age-associated factors. Dachshunds may represent another high-risk group, in which long and crowded dentition, combined with breed-specific genetic factors, contribute to both a higher baseline risk and a steep age-related increase. Sighthounds were likewise classified into a pattern characterized by both a high baseline risk and a steep age-related increase. Therefore, these two distinct groups are expected to have a particularly high lifetime risk for periodontal disease. Greyhounds, which belong to the sighthound group, have repeatedly been reported as high-risk breeds, despite the general trend that small breeds tend to exhibit higher periodontal disease susceptibility (20, 37). This may indicate that the high-risk and strong age-effect pattern observed for the sighthound group was consistently detected because this group inherently reflects such breed-specific characteristics. These findings imply that even breeds traditionally considered to be at low risk may require dental care equivalent to that required by high-risk breeds if they exhibit steep age-related increases in risk.

However, several breeds deviated from general trends in their respective groups. For example, the French Bulldog, classified within the companion group (9G), which is generally characterized by high baseline and moderate age-related risk, showed an exceptionally low baseline risk (79th of 81 breeds) but a markedly high age-related effect (4th of 81 breeds). Similarly, the Siberian Husky, belonging to the spitz group (5G), showed a low baseline rank (70th out of 81 breeds) but one of the highest age-related effects (5th out of 81 breeds). Such inconsistencies indicate that morphological, genetic, or behavioral factors unique to each breed may modulate the risk of periodontal disease beyond group-level characteristics. The specific traits responsible for these differences are unclear. One possible approach to identify such determinants is to focus on breeds that share similar baseline risks but show markedly different age-related effects. For example, the Bichon Frise and Kaninchen Dachshund exhibited the two strongest age-related effects, whereas the Yorkshire Terrier and Papillon showed the weakest effects despite having comparable baseline risks. Such contrasting breeds may help elucidate the biological mechanisms underlying age-related periodontal deterioration, including connective tissue fragility, immune senescence, and age-associated changes in the oral microbiota, as well as the genetic and morphological traits that influence these processes.

### 4.6 Breed-level patterns in cats

In cats, differences were observed only in age-related effects, whereas baseline risk showed minimal variation among breeds. The annual age effect ranged from an OR of 1.144 in Siamese cats to 1.056 in Russian Blues, indicating a nearly 9% difference that could result in a substantial divergence in prevalence with advancing age. Girard et al. (18) and O’Neill et al. (9) reported higher risks in certain breeds, whereas Lommer and Verstraete (17) and Mestrinho et al. (23) reported no significant breed effects. In the present study, baseline risk did not differ among breeds, but a significant variation was detected in the age effect. In dogs, baseline risk is strongly influenced by body size and structural diversity among breeds; however, these factors may be less variable in cats or may have a smaller impact on periodontal risk. O’Neill et al. (9) also noted that breed-related differences in periodontal disease risk were smaller in cats than in dogs. Among breeds previously identified as predisposed to periodontal disease, Siamese, Somali, and Persian cats ranked 1st, 6th, and 7th, respectively, out of 38 breeds for the age effect, suggesting that their previously reported high risk may reflect stronger age-related increases. Conversely, Maine Coons and Bengals ranked 20th and 35th, respectively, indicating that these breeds may maintain intermediate or lower risks even at older ages. Brachycephalic breeds tend to exhibit relatively high age effects, which may be attributable to the craniofacial conformation or occlusal morphology, which increases mechanical stress on the dentition. In particular, Exotic Shorthair, Exotic, Himalayan, and Persian cats ranked 2nd, 3rd, 4th, and 7th, respectively, for the age effect, highlighting a consistent trend among short-headed breeds. These findings suggest that cat breeds with stronger age-related effects may experience an earlier onset or faster progression of periodontal disease. Therefore, these breeds would benefit from regular oral examinations and preventive dental care at a young age.

### 4.7 Limitations and future directions

This study used a nationwide insurance claims database in Japan to comprehensively evaluate the risk of periodontal disease according to age, body size, breed group, and individual breeds. The use of large-scale, real-world clinical data is a major strength of this study; however, several limitations should be noted. First, the insurance claims data did not include information on disease stage or severity, making it impossible to distinguish between gingivitis and advanced periodontitis. Marshall et al. (21) reported that the progression of periodontal disease accelerates with age in Miniature Schnauzers, underscoring the need for longitudinal stage-specific analyses to assess disease progression. Second, environmental and behavioral factors such as housing conditions, feeding practices, and oral care habits were not considered. For example, soft diets have been associated with an increased risk of periodontal disease (6), and the presence or absence of home oral care has been correlated with oral health status in cats and dogs (38). These variables may influence the onset and progression of periodontal disease, and future studies should incorporate questionnaire-based or behavioral data to complement clinical information. Third, although sex was a significant factor in both dogs and cats, information on the neuter or spay status was not available. In cats, neutered cats were reported to exhibit higher odds of developing periodontal disease than were intact cats (9). Therefore, a potential bias related to reproductive status cannot be ruled out, and the observed sex effects may not accurately reflect true biological differences. In addition, a Japanese study demonstrated that the diversity of periodontal disease–associated bacteria increased with age in dogs (32). This finding suggests that age-related changes in oral microbiota and immune senescence may partially explain the differences in age-related effects observed among breed groups or individual breeds. Future studies that integrate longitudinal assessments of disease progression with microbiological and immunological analyses are essential for a more precise understanding of periodontal disease dynamics and risk stratification. Overall, the present findings highlight the importance of preventive dental care strategies tailored to age and breed characteristics and underscore the need for early diagnosis and individualized oral management in veterinary practice.

## Data Availability

The dataset contains proprietary and confidential insurance claim records and cannot be shared publicly. Access to the data is restricted by the data provider (Anicom Insurance Inc.) and can be granted only upon reasonable request and with appropriate data-use agreements. Requests to access these datasets should be directed to Ryo Horie, ryo.horie@ani-com.com.

## References

[1] Niemiec BA. Periodontitis In: Veterinary periodontology. West Sussex, UK: John Wiley & Sons, Inc (2013). 51–68.

[2] Bellows J Berg ML Dennis S Harvey R Lobprise HB Snyder CJ . 2019 AAHA dental care guidelines for dogs and cats. J Am Anim Hosp Assoc. (2019) 55:49–69. doi: 10.5326/JAAHA-MS-6933, 30776257

[3] Ruparell A Wallis C Haydock R Cawthrow A Holcombe LJ. Comparison of subgingival and gingival margin plaque microbiota from dogs with healthy gingiva and early periodontal disease. Res Vet Sci. (2021) 136:396–407. doi: 10.1016/j.rvsc.2021.01.011, 33799170

[4] Harvey CE. Management of periodontal disease: understanding the options. Vet Clin North Am Small Anim Pract. (2005) 35:819–36, vi, vi. doi: 10.1016/j.cvsm.2005.03.002, 15979515

[5] Wallis C Holcombe LJ. A review of the frequency and impact of periodontal disease in dogs. J Small Anim Pract. (2020) 61:529–40. doi: 10.1111/jsap.13218, 32955734

[6] Lund EM Armstrong PJ Kirk CA Kolar LM Klausner JS. Health status and population characteristics of dogs and cats examined at private veterinary practices in the United States. J Am Vet Med Assoc. (1999) 214:1336–41. doi: 10.2460/javma.1999.214.09.1336, 10319174

[7] O Neill DG Church DB McGreevy PD Thomson PC Brodbelt DC. Prevalence of disorders recorded in dogs attending primary-care veterinary practices in England. PLoS One. (2014) 9:e90501. doi: 10.1371/journal.pone.0090501, 24594665 PMC3942437

[8] Robinson NJ Brennan ML Cobb M Dean RS. Investigating preventive-medicine consultations in first-opinion small-animal practice in the United Kingdom using direct observation. Prev Vet Med. (2016) 124:69–77. doi: 10.1016/j.prevetmed.2015.12.010, 26775818

[9] O’Neill DG Blenkarn A Brodbelt DC Church DB Freeman A. Periodontal disease in cats under primary veterinary care in the UK: frequency and risk factors. J Feline Med Surg. (2023) 25:1098612X231158154. doi: 10.1177/1098612x231158154, 36912667 PMC10812011

[10] DeBowes LJ Mosier D Logan E Harvey CE Lowry S Richardson DC. Association of periodontal disease and histologic lesions in multiple organs from 45 dogs. J Vet Dent. (1996) 13:57–60. doi: 10.1177/089875649601300201 9520780

[11] Glickman LT Glickman NW Moore GE Goldstein GS Lewis HB. Evaluation of the risk of endocarditis and other cardiovascular events on the basis of the severity of periodontal disease in dogs. J Am Vet Med Assoc. (2009) 234:486–94. doi: 10.2460/javma.234.4.486, 19222358

[12] Soe E Davison J Süld K Valdmann H Laurimaa L Saarma U. Europe-wide biogeographical patterns in the diet of an ecologically and epidemiologically important mesopredator, the red fox *Vulpes vulpes*: a quantitative review. Mamm Rev. (2017) 47:198–211. doi: 10.1111/mam.12092

[13] Trevejo RT Lefebvre SL Yang M Rhoads C Goldstein G Lund EM. Survival analysis to evaluate associations between periodontal disease and the risk of development of chronic azotemic kidney disease in cats evaluated at primary care veterinary hospitals. J Am Vet Med Assoc. (2018) 252:710–20. doi: 10.2460/javma.252.6.710, 29504859

[14] Summers JF O’Neill DG Church D Collins L Sargan D Brodbelt DC. Health-related welfare prioritisation of canine disorders using electronic health records in primary care practice in the UK. BMC Vet Res. (2019) 15:163. doi: 10.1186/s12917-019-1902-0, 31118035 PMC6532203

[15] Silva C Requicha J Dias I Bastos E Viegas C. Genomic medicine in canine periodontal disease: a systematic review. Animals (Basel). (2023) 13:2463. doi: 10.3390/ani13152463 37570272 PMC10417655

[16] Gengler W Dubielzig R Ramer J. Physical examination and radiographic analysis to detect dental and mandibular bone resorption in cats: a study of 81 cases from necropsy. J Vet Dent. (1995) 12:97–100. doi: 10.1177/089875649501200301, 9693633

[17] Lommer MJ Verstraete FJ. Radiographic patterns of periodontitis in cats: 147 cases (1998-1999). J Am Vet Med Assoc. (2001) 218:230–4. doi: 10.2460/javma.2001.218.230, 11195829

[18] Girard N Servet E Biourge V Hennet P. Periodontal health status in a colony of 109 cats. J Vet Dent. (2009) 26:147–55. doi: 10.1177/089875640902600301, 19950514

[19] Kortegaard HE Eriksen T Baelum V. Periodontal disease in research beagle dogs--an epidemiological study. J Small Anim Pract. (2008) 49:610–6. doi: 10.1111/j.1748-5827.2008.00609.x, 18793256

[20] O’Neill DG Mitchell CE Humphrey J Church DB Brodbelt DC Pegram C. Epidemiology of periodontal disease in dogs in the UK primary-care veterinary setting. J Small Anim Pract. (2021) 62:1051–61. doi: 10.1111/jsap.13405, 34374104 PMC9291557

[21] Marshall MD Wallis CV Milella L Colyer A Tweedie AD Harris S. A longitudinal assessment of periodontal disease in 52 miniature schnauzers. BMC Vet Res. (2014) 10:166. 25179569 10.1186/1746-6148-10-166 PMC4236762

[22] Harvey CE Shofer FS Laster L. Association of age and body weight with periodontal disease in north American dogs. J Vet Dent. (1994) 11:94–105. doi: 10.1177/089875649401100301, 9693607

[23] Mestrinho LA Louro JM Gordo IS Niza MMRE Requicha JF Force JG . Oral and dental anomalies in purebred, brachycephalic Persian and exotic cats. J Am Vet Med Assoc. (2018) 253:66–72. doi: 10.2460/javma.253.1.66, 29911947

[24] Niemiec BA. Oral pathology. Top Companion Anim Med. (2008) 23:59–71. doi: 10.1053/j.tcam.2008.02.002, 18482706

[25] Anicom Holdings I. White paper on household animals 2024 Anicom (2024).

[26] Bates D Mächler M Bolker B Walker S. Fitting linear mixed-effects models using lme4. arXiv [statCO]. (2014)

[27] Villanueva RAM Chen ZJ. ggplot2: elegant graphics for data analysis (2nd ed.). Meas Interdiscip Res Perspect. (2019) 17:160–7. doi: 10.1080/15366367.2019.1565254

[28] Auguie B Antonov A. gridExtra: Miscellaneous functions for" grid" graphics. R package version 2.3. 2007.

[29] Wilke C. Cowplot: Streamlined plot theme and plot annotations for “ggplot2”. R package version 1.2.0 2025.

[30] Wickham H Pedersen TL Seidel D Scales: Scale functions for visualization. R package version 1.4.0 2025

[31] Lenth RV Piaskowski J Emmeans: Estimated marginal means, aka least-squares means. R package version 2.0.0 2025

[32] Hirai N Shirai M Kato Y Murakami M Nomura R Yamasaki Y . Correlation of age with distribution of periodontitis-related bacteria in Japanese dogs. J Vet Med Sci. (2013) 75:999–1001. doi: 10.1292/jvms.13-0041, 23485527

[33] Japan Pet Food Association. (2024). National Survey on dog and cat ownership in 2024 [internet]. National Survey on Dog and Cat Ownership. Available online at: https://petfood.or.jp/data-chart/

[34] Lăzureanu PC Popescu F Tudor A Stef L Negru AG Mihăilă R. Saliva pH and flow rate in patients with periodontal disease and associated cardiovascular disease. Med Sci Monit. (2021) 27:e931362. doi: 10.12659/msm.931362 34305133 PMC8323473

[35] Koppolu P Sirisha S Penala S Reddy PK Alotaibi DH Abusalim GS . Correlation of blood and salivary pH levels in healthy, gingivitis, and periodontitis patients before and after non-surgical periodontal therapy. Diagnostics. (2022) 12:97. doi: 10.3390/diagnostics12010097, 35054264 PMC8774853

[36] Bradshaw JWS Casey RA Brown SL. Feeding behaviour In: The behaviour of the domestic cat. UK: CABI (2012). 113–27.

[37] O’Neill DG Rooney NJ Brock C Church DB Brodbelt DC Pegram C. Greyhounds under general veterinary care in the UK during 2016: demography and common disorders. Canine Genet Epidemiol. (2019) 6:4. doi: 10.1186/s40575-019-0072-5 31179010 PMC6547581

[38] Buckley C Colyer A Skrzywanek M Jodkowska K Kurski G Gawor J . The impact of home-prepared diets and home oral hygiene on oral health in cats and dogs. Br J Nutr. (2011) 106:S124–7. doi: 10.1017/S0007114511000821 22005407

