# Supplementary material for: Species- and breed-associated heterogeneity in age-related increases in periodontal disease risk among dogs and cats based on Japanese insurance claim data
Source: Front Vet Sci. 2026 Feb 10;13:1764413. doi: 10.3389/fvets.2026.1764413 (PMC12930463; doi:10.3389/fvets.2026.1764413)
Supplement: Supplementary file 1 [file Data_Sheet_1.docx]

Supplementary Material

# Supplementary Figures and Tables

**
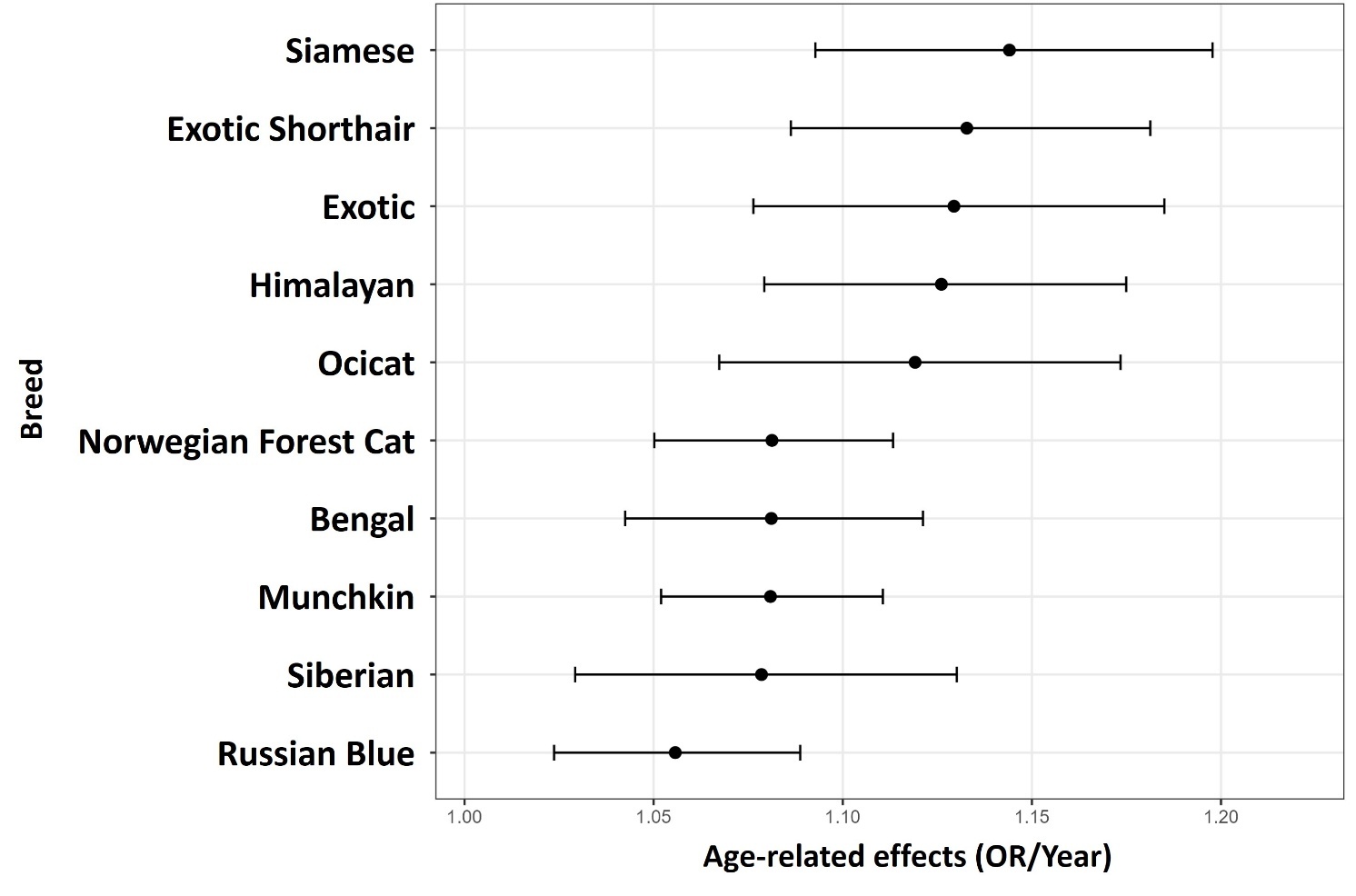
**

**Figure S1.** Top five and bottom five cat breeds in age-related effect

The five breeds with the strongest and weakest age-related effects are shown, based on breed-specific estimates. The baseline risk was estimated to have negligible variance across breeds in the model. The results for all other breeds are provided in Table S4. Error bars indicate 95% confidence intervals.

**Table S1.** Summary of insurance claims by year and dog breed

See Table_S1.xlsx

Start_Year indicates the policy start year, where N_total represents all insured animals, N_accident represents animals with at least one claim, and N_PD represents animals with periodontal disease-related claims. Male% indicates the proportion of males among all insured animals.

**Table S2.** Summary of insurance claims by year and cat breed

See Table_S2.xlsx

Start_Year indicates the policy start year, where N_total represents all insured animals, N_accident represents animals with at least one claim, and N_PD represents animals with periodontal disease-related claims. Male% indicates the proportion of males among all insured animals.

**Table S3.** Distribution of diagnostic terms included in the periodontal disease category by species and policy start year

See Table_S3.xlsx

Start_Year indicates the policy start year, Diagnostic_term indicates each minor diagnostic term included in the periodontal disease definition, and N_animals represents the number of animals with at least one insurance claim containing the corresponding diagnostic term. Diagnostic terms are not mutually exclusive; therefore, a single animal may be counted under more than one diagnostic term.

**Table S4.** Breed-specific baseline risk and age-related effect in dogs

See Table_S4.xlsx

N_total represents all insured animals, and N_PD represents animals with periodontal disease–related claims. base_prob denotes baseline risk, and base_L95 and base_U95 indicate the lower and upper bounds, respectively, of the 95% confidence interval for baseline risk. OR_per_year denotes the age-related effect, and OR_L95 and OR_U95 indicate the lower and upper bounds of the 95% confidence interval.

**Table S5.** Breed-specific baseline risk and age-related effect in cats

See Table_S5.xlsx

N_total represents all insured animals, and N_PD represents animals with periodontal disease–related claims. base_prob denotes baseline risk, and base_L95 and base_U95 indicate the lower and upper bounds, respectively, of the 95% confidence interval for baseline risk. OR_per_year denotes the age-related effect, and OR_L95 and OR_U95 indicate the lower and upper bounds, respectively, of the 95% confidence interval.
